# Interview with Prof. Volker Hömberg, President-Elect of the World Federation for Neurorehabilitation Societies (WFNR)

**DOI:** 10.25122/jml-2022-1029

**Published:** 2022-10

**Authors:** Alexandra Gherman, Andreea Doria Constantinescu, Dafin Fior Muresanu

**Affiliations:** 1 RoNeuro Institute for Neurological Research and Diagnostic, Cluj-Napoca, Romania; 2 Department of Neuroscience, Iuliu Hatieganu University of Medicine and Pharmacy, Cluj-Napoca, Romania

**Interviewer: Andreea Doria Constantinescu**

**Interviewee: Prof. Volker Hömberg**

Prof. **Volker Hömberg** currently serves as President-Elect of the World Federation for Neurorehabilitation Societies (WFNR) and Vice-President of the European Federation of Neurorehabilitation Societies (EFNR).

This is an adapted interview from the 12^th^ Teaching Course on Neurorehabilitation (September 2^nd^–3^rd^, 2022, Eforie Nord, Romania) and the WFNS Symposium on CEREBROVASCULAR DISEASE – A Multidisciplinary Approach (September 8^th^–10^th^, 2022, Cluj-Napoca, Romania).

**A.D.C.:** Hello, Professor Hömberg! Thank you very much for your presence in these important scientific events in [these] days in Romania – the 12^th^ Teaching Course on Neurorehabilitation in Eforie and now, in Cluj-Napoca, the Symposium on Cerebrovascular Disease – A Multidisciplinary Approach. How relevant and useful is this multidisciplinary approach in the field of cerebrovascular diseases?

**V.H.:** Well, a multidisciplinary approach is, in general, very important. In all fields of medicine, you cannot do anything without multidisciplinary approach. Also in cerebrovascular diseases or stroke, there is an interaction; with multiple stakeholders involved there is the general practitioner, the acute neurologist, the neurosurgeon, the intensivist, and, later on, in rehabilitation, you have the rehabilitation neurologist, you have the physical therapist, the occupational therapist, and so forth. And the entire, let's say, piece of art, what to do with a stroke patient, is very much dependent on the interplay of all the disciplines involved. Therefore, a multidisciplinary is an absolute must. On the other side, some people claim multidisciplinarity and it's not always true. On these meetings here, in Romania, for years, we have always tried to keep them as multidisciplinary as possible. The result was that we were not just pretending to do so, but that happened in actual fact.

**A.D.C.:** Which is great! In your opinion, are there any particular aspects that scientists and practitioners should contemplate more in order to better embrace the continuous evolution of digitalization?

**V.H.:** Well, that is a very, very important and interesting question. I think, over the last years, we learned to have greater ease in using digital technology worldwide for many purposes. In the field I am working in, which is neighbouring to many other fields in medicine and neurology, in particular, I see three major points. One is the use of digital technologies in communications. That is very important, for instance, when it comes to spreading information, knowledge, skills and procedures around the world. We have hundreds of millions of people around the world who have no access whatsoever to any sort of decent rehabilitation. There is no chance that they will have it in not too distant future. In this gap, digital technology, of course, can be extremely, extremely helpful. The second element, of importance in our fields and in many other fields of medicine as well, is the use of digital technology to improve the contents of what we are doing. For instance, using augmented or virtual reality to improve the way we are presenting, let's say, gaming material, stimulation material, or task material to patients is improving dramatically. For instance, in the field of brain-computer interfaces, picking up commands from the brain to make, just by thinking, things happen or exoskeletons or the patients' limbs move, opens new therapeutic windows. Years ago, it took hours to train a patient to control an exoskeleton, whereas today, with the most advanced brain-computer interfaces, it takes just minutes. This is, of course, offering completely new perspectives using digital technologies. The third element I would like to mention is the use of artificial intelligence to improve the quality of clinical decision making. For clinical and research purposes digital technologies open completely new spaces and opportunities. Nobody can read all of the relevant information published regularly, but an artificial intelligence system, of course, can take care of this. And if you have, let's say, a young doctor in a hospital faced with an interesting or complex patient, artificial intelligence certainly can help him find the proper diagnosis and treatment paths. In this sense, artificial intelligence can help physicians very much to make decisions. Furthermore, we have been involved in many sorts of studies in organizing knowledge, making or deriving guidelines from these studies, which is a very time-consuming work [and] many, many scientists are involved in that, and I could imagine just to update this information for the future, artificial intelligence systems can be helpful to do it on a, let's say, monthly or even weekly basis.

**A.D.C.:** Alright, and one last question. As the world enters the third year of the COVID-19 pandemic, physicians are facing complex neurorehabilitation cases. In your opinion, what improvements are needed in the diagnosis, treatments, and of course, follow-up of post-COVID neurological patients?

**V.H.:** Well, we certainly are faced with a situation [that] there is something existing as long COVID problems. My impression is that this long COVID problem sometimes is a little bit overemphasized, even creating a lot of anxiety and fear in many patients who definitely do not have long COVID problems. So, it really needs careful analysis. It's interesting to know [that] there are many varieties of acute and long COVID problems. The interesting finding is that people who have suffered a lot from acute CORONA-19 infection, with a long stay in the ICU, hardly ever develop these long COVID problems. They may have longer-lasting neuromuscular problems, as many people staying in ICUs for a long time have what we call critical illness neuromyopathies. But those patients who have only mild CORONA problems have a higher tendency to develop the typical fatigue and cognitive problem with long COVID. And this long COVID fatigue, which is very similar to what we know for many years as chronic fatigue syndrome, for instance, is a particular problem. We believe, more and more, that it may be an autoimmune disease, with similar examples: in 1955, at the Royal Free Hospital in London, they had an outbreak of what probably was a viral infection, and about a hundred or so from the staff at the Royal Free, were affected with a strange type of disorder, with a lot of fatigue problems involved. The term "chronic fatigue" was coined later, but this was probably the first of these outbreaks. We have to consider that long COVID problem related – fatigue may be an autoimmune disease. We have seen similar clinical presentations also with SARS (severe acute respiratory syndrome) and MERS (Middle East Respiratory Syndrome) and other viral infections. Nevertheless, I think we need to learn more about that. We certainly also need to learn more about possible, let's say, interventions for long COVID problems. At the horizon, there may be one or the other drug which may be helpful. Some people are playing with plasmapheresis/aphaeresis systems to get rid of possible antibodies in the blood, as a putative cure. My impression is that many of the patients suffering from fatigue-dominated long COVID problems certainly need help. We have developed a special app for counselling patients with long COVID problems; this is available in 11 languages now, worldwide, free of charge, and can be helpful for people to restructure their everyday life, to have sort of a fatigue diary and also having some hints for relaxation techniques and for, let's say, moderate if any, moderate exercising. The greatest problem for these people is that they may eventually do too much exercise. Because people believe exercise is good for rehabilitation, but that is, eventually, not good for fatigue-dominated long COVID patients. So, we certainly have to learn more about that in the future.

**A.D.C.:** Great! Thank you so much for sharing your opinions and great experience!

**V.H.:** My pleasure! Thank you very much!

**A.D.C.:** [We are] looking forward to seeing you at our future events!

**V.H.:** It's a great pleasure to be back in Romania after these years, and the last days certainly were a great pleasure for me. Thank you!

**A.D.C.:** Thank you!

